# Bilateral septic arthritis with rapid progressive destruction of the femoral head after joint injection in rheumatoid arthritis

**DOI:** 10.5194/jbji-6-255-2021

**Published:** 2021-07-07

**Authors:** Viola Freigang, Florian Baumann, Volker Alt

**Affiliations:** Department of Trauma Surgery, Regensburg University Medical Center, 93042 Regensburg, Germany

## Abstract

This report is on a 61-year-old patient with steroid therapy for rheumatoid arthritis and pain in the groin on both sides who got injections with hyaluronic acid in both hip joints. After 12 weeks the X-ray of the pelvis showed rapid progressive destruction of both hip joints.

We report on a clinical case of rapid progressive destruction of the femoral head caused by infection after joint injection in rheumatoid arthritis.

This report is on a 61-year-old patient with steroid therapy for rheumatoid arthritis and pain in the groin on both sides. Twelve weeks before the first consultation in our institution, an orthopedic surgeon revealed the diagnosis of osteoarthritis of the hip on both sides (Fig. 1a) and injected hyaluronic acid in both hip joints. Subsequently, the pain increased, leading to immobility and poor general condition. Twelve weeks after the injection, the patient presented to our emergency department with fever, reduced general condition and pain in both hips.

**Figure 1:**
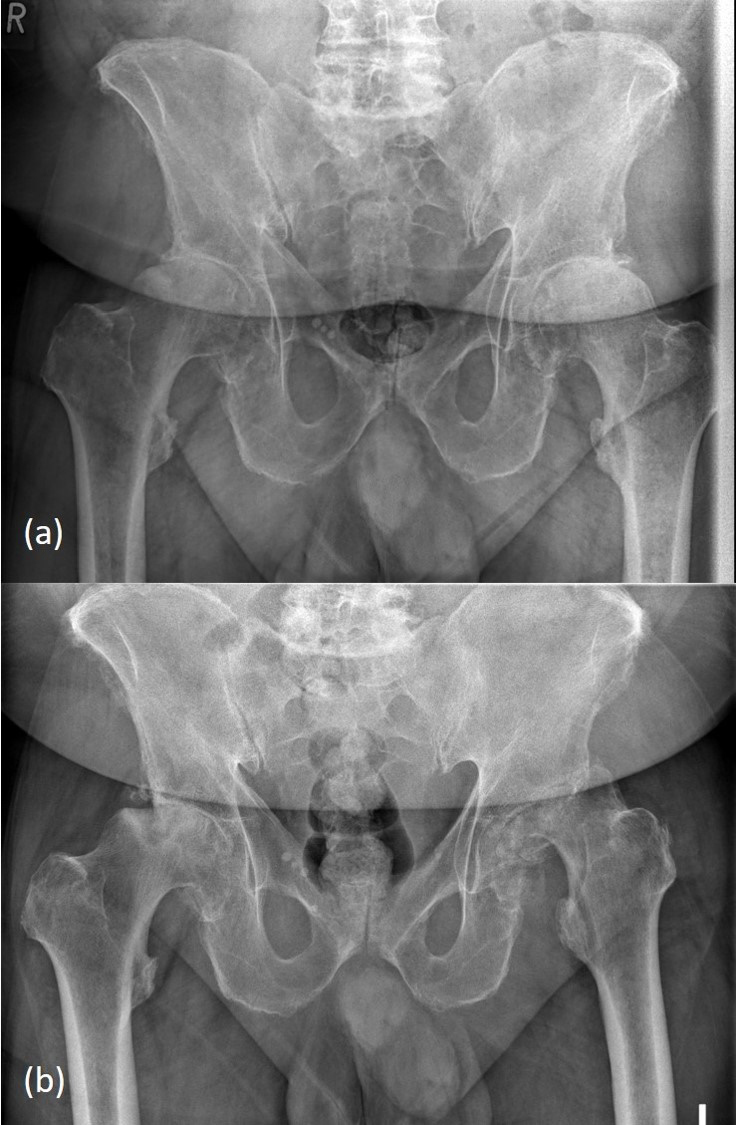
Copyright is held by the Regensburg University Medical Center. **(a)** X-ray of the pelvis showing advanced osteoarthritis of both hip joints. **(b)** X-ray of the pelvis showing femoral head necrosis on both sides with extensive osteolysis of both femoral heads and bone loss of the acetabular rim on the left side.

On admission, the patient's CRP level was 371 mg/L and the WBC count was 17.3 per nanolitre. The X-ray of the pelvis shows rapid progressive destruction of both hip joints with extensive osteolysis, a subluxation and an acetabular bony defect of the left side (Fig. 1b). The patient received emergent surgical debridement of both hip joints and implantation of antibiotic-coated articular spacers. After treatment of the infection with repeated debridement and spacer exchange, we implanted a silver-coated non-cemented standard prosthesis on both sides.

We recommend a strict risk–benefit analysis before intra-articular application of hyaluronic acid in patients with rheumatoid arthritis and long-term steroid therapy.

## Data Availability

No data sets were used in this article.

